# Canine brain imaging with a new low-field portable (0.05 T) MRI scanner: a pilot *in vivo* comparison to conventional 1.5 T

**DOI:** 10.1080/01652176.2026.2696026

**Published:** 2026-07-14

**Authors:** Elisabeth Maria Burgers, Samantha Miles, Ruben van den Broek, Chloé Najac, Beatrice Lena, Thomas O’Reilly, Andrew Webb, Stefanie Veraa

**Affiliations:** a Department of Clinical Sciences, Division of Diagnostic Imaging, Utrecht University, Utrecht, the Netherlands; b IDEXX Telemedicine Consultants, Hoofddorp, the Netherlands; c Department of Radiology, C.J. Gorter MRI Center, Leiden University Medical Center, Leiden, the Netherlands

**Keywords:** Canine neuroimaging, low field magnetic resonance imaging, image contrast, visual grading characteristic analysis, hydrocephalus, sustainable imaging, permanent magnets, Halbach array, one health

## Abstract

Prohibitive costs associated with high-field MRI systems have resulted in reduced access in resource-limited areas with consequential healthcare inequities. This has renewed interest in purposefully designed low-field (LF) systems that, despite inherently lower signal-to-noise ratios, have advantages in terms of cost, portability, and accessibility. This pilot study successfully translated a previously developed LF (0.05 T) MRI canine cadaver brain protocol for *in vivo* application and compared the acquired images with paired 1.5 T images from 21 different canine patients. The visibility of 15 anatomic features was ordinarily scored (scale 1–3) on transverse T1-weighted post-contrast (21/21 patients) and T2-weighted (17/21 patients) sequences. Lateral ventricles were easily visualized (89.3%–100% scored 3/3) across all sequence‒system combinations, with T2-weighted sequences also providing good identification of the mesencephalic aqueduct (56.3%–100%), fourth ventricle (77.1%–100%), and thalamus (68.8%–100%). Absolute visual grading characteristics (VGC) analysis confirmed comparable performance of the LF system to the 1.5 T MRI for these features, a particularly relevant finding given the system's originally intended application for pediatric hydrocephalus neuroimaging in resource-limited areas. This study represents the first *in vivo* usage and evaluation of a 0.05 T MRI in a clinical veterinary context.

## 1. Introduction

Magnetic resonance imaging (MRI) is an indispensable imaging modality, the clinical usage of which continues to grow in both veterinary and human medicine (Kang et al. 2009; Gavin, 2011; Hespel and Cole 2018; Jacqmot et al. 2022; Anon 2023). Relative to computed tomography (CT), one of the main benefits of MRI is its superior soft-tissue contrast. This is particularly useful for evaluation of the brain and spinal cord, making this modality indispensable in the diagnostic evaluation of any patient presenting with neurological signs (Kang et al. 2009; Hespel and Cole 2018; Jacqmot et al. 2022). The diagnostic capabilities of an MRI are largely dictated by its magnetic field strength, expressed in Tesla (T), with systems broadly defined as low-field (LF; < 0.1 T), mid-field (0.2–1.4 T), high-field (HF; 1.5–3 T), and ultra-high field (>7 T) (Hespel and Cole 2018; Geethanath and Vaughan 2019; Jacqmot et al. 2022). An increasing number of HF MRI systems are available in veterinary medicine, with numerous veterinary studies in the last decade that evaluate 3 T and 7 T imaging (Kang et al. 2009; Konar and Lang 2011; Anaya García et al. 2015; Jacqmot et al. 2017; Hespel and Cole 2018; Czeibert et al. 2019; Nitzsche et al. 2019; Johnson et al. 2020; Jacqmot et al. 2022). A critical factor driving the investment in higher field strengths for general clinical usage is the broader diagnostic capabilities associated with the improved signal-to-noise ratio (SNR) seen with increasing field strength. As SNR is intrinsically linked with spatial resolution, a finer spatial resolution is achievable as SNR increases. As spatial resolution improves, so does the ability to localize and characterize a variety of lesions (Kang et al. 2009; Hespel and Cole 2018; Geethanath and Vaughan 2019; Marques et al. 2019; Sarracanie and Salameh 2020; Wald et al. 2020; Jacqmot et al. 2022).

However, commercial HF systems are expensive due to the combination of the purchasing costs of the system itself, approximately $1 million per Tesla, and costs related to installation and maintenance contracts. These prohibitive costs have created a healthcare inequity with resource-limited areas having reduced access to MRI (Konar and Lang 2011; Hespel and Cole 2018; Geethanath and Vaughan 2019; Marques et al. 2019; O’Reilly et al. 2019; Sarracanie and Salameh 2020; Wald et al. 2020). In human medicine, this has renewed interest in the development of clinically applicable LF MRI systems to improve accessibility to this modality. Various technological advancements, in particular related to system design and processing algorithms, have helped to compensate for the inherently lower SNR found in low-field systems (Geethanath and Vaughan 2019; Marques et al. 2019; O’Reilly et al. 2019; Sarracanie and Salameh 2020; Wald et al. 2020; O’Reilly et al. 2021; de Vos et al. 2021; Obungoloch et al. 2023; Anoardo and Rodriguez 2023; Kravchenko et al. 2025). While it should be emphasized that LF and portable MRI remain in the early stages of development, a number of recent reviews highlight the potential clinical applications in humans, such as for the assessment of gross intracranial pathologies or strokes (Arnold et al. 2023; Balaji et al. 2024; Chen et al. 2025; Gagliardo et al. 2025; Samardzija et al. 2026; Joshi et al. 2026).

One such system is a portable, low-cost, and open-source 0.05 T MRI developed by O’Reilly et al. for human pediatric neuroimaging, specifically hydrocephalus, in resource-limited regions (O’Reilly et al. 2019; O’Reilly et al. 2021; de Vos et al. 2021; Obungoloch et al. 2023). A preliminary assessment of this system in veterinary patients was performed by the current authors in an unpublished pilot cadaver study. By scanning a diverse population of 23 canine cadavers, a brain protocol was developed with subsequent comparison of the 0.05 T images with paired sequences acquired with a 1.5 T MRI. The results showed that a basic brain scan protocol, consisting of transverse T1w and T2w turbo spin echo (TSE) sequences, is feasible with reasonable data-acquisition times (mean T1w 05:16 min, T2w 09:16 min). The 0.05 T images showed adequate reproduction of the ventricular system and suggested that additional features could be detected more reliably, provided there was consistent image quality. Due to the cadaveric nature of the study, post-mortem artifacts limited the evaluation of various anatomic features. Furthermore, data acquisition was not representative of a true clinical environment where the risk of other artifacts, such as electromagnetic interference (EMI) from anesthetic monitoring equipment, can diminish image quality.

Our aim in the current study was to further evaluate the previously described LF MRI developed by O’Reilly et al. by *in vivo* assessment of a diverse population of canine patients (O’Reilly et al. 2019; O’Reilly et al. 2021; de Vos et al. 2021; Obungoloch et al. 2023). The objectives of the study were: (1) translation and optimization of the previously developed canine brain LF MRI protocol for *in vivo* application, and (2) exploratory image evaluation by comparison of LF and HF (1.5 T) images.

## 2. Materials and methods

### 2.1. Case selection

This pilot study was performed at the primary author’s institution (Faculty of Veterinary Medicine, Utrecht University) and approved by the Animal Welfare Body Utrecht under work protocol no: 10813-2023-01.

Client-owned dogs with a clinical indication for a diagnostic brain MRI were prospectively recruited over a one-month period from May to June 2024. Clinical indications included re-evaluation of a previously diagnosed intra-cranial lesion or neurological signs localized to the neurocranium. Additional inclusion criteria were small- to medium-sized dogs with a maximum head diameter of ≤14  cm and American Society of Anesthesiologists (ASA) status ≤3 as determined by a board-certified Diplomate of the European College of Veterinary Anesthesia and Analgesia, ECVAA. Patient age, sex, and specific breed were not considered within the inclusion criteria. Exclusion criteria were patients lacking paired HF and LF acquired sequences. Case inclusion or exclusion was decided by a second-year European College of Veterinary Diagnostic Imaging (ECVDI) resident in veterinary diagnostic imaging (E.B.) under the supervision of a board-certified Diplomate of the ECVDI (S.V.).

The following clinical information was recorded from the medical records of all included patients: signalment (age at the time of imaging, breed, sex, castration status), body weight at the time of imaging, ASA status at the time of imaging, provided clinical history in the imaging request, and the final imaging diagnosis from the official radiological report. The report was written by the supervising clinical radiologist on the day of scanning after assessment of all HF sequences acquired according to the standardized clinical protocol at the primary author’s institution.

### 2.2. Anesthetic protocol

All scans were performed over a two-week period in June 2024. After pre-anesthetic evaluation, patients were anesthetized according to non-standardized protocols determined on a case-by-case basis by the supervising board-certified veterinary anesthesiologist. All patients were maintained under inhalational anesthesia with isoflurane (isoflurane 1000 mg/g, Isoflutek®, Alivira, Barcelona, Spain). During the HF scans, MRI-compatible anesthetic monitoring equipment was used (Expression Model MR400, Phillips Healthcare, Eindhoven, the Netherlands). During LF scans, non-MRI compatible equipment was utilized, including capnography, non-invasive blood pressure, and pulse oximeter (BeneView T5, Mindray, Haryana, India; Pettrust non-invasive blood pressure monitor, Biocare, Taoyuan City, Taiwan; UT100V veterinary pulse oximeter, UTech Co., Chongqing, China).

### 2.3. Image acquisition

#### 2.3.1. HF (high-field) MRI

All patients first underwent a brain scan in sternal recumbency with the 1.5 T MRI (Ingenia, Philips Healthcare, Best, the Netherlands) according to the standardized clinical protocol at the primary author’s institution. This protocol includes post-contrast sequences acquired 1 min after intravenous administration of gadoterate meglumine (0.15 mmol/kg, Dotarem 0.5 mmol/mL, Guerbet, Gorinchem, the Netherlands). For the purpose of this study, the only sequences evaluated are the pre-contrast transverse 2D multi-slice T2-weighted (T2w) turbo spin-echo (TSE) and post-contrast transverse T1-weighted (T1w) 2D multi-slice spin echo (SE) sequences, from this point forward referred to as T2w and T1w, respectively. It was elected to evaluate the post-contrast T1w sequences, as the subsequently acquired LF sequences would technically be late-phase post-contrast, as all scanning was performed under one anesthetic session in accordance with the ethical work protocol. Additionally, this same protocol did not allow for re-administration of gadoterate meglumine for the LF sequences, representing an inherent methodological limitation related to suboptimal contrast enhancement in these sequences due to the reduced relaxivity of gadolinium at lower field strengths (Konar and Lang 2011; Hori et al. 2021; Chen et al. 2025).

#### 2.3.2. LF (low-field) MRI

**Figure 1.**
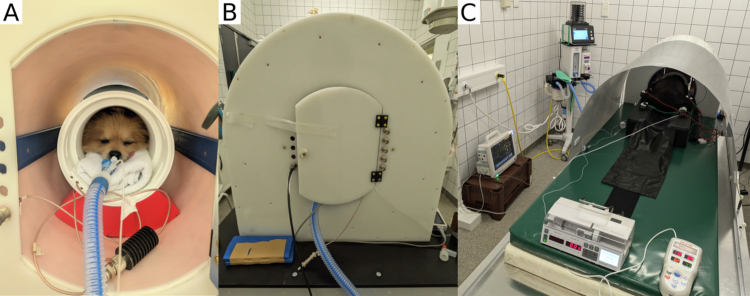
(A) Back of the bore with the small door open showing the positioning of patient #15 within the elliptical spiral-solenoid transmit/receive head coil. (B) Back of the bore with the door closed, as much as possible, to allow access for the anesthetic re-breathing tube. (C) Front of the bore with the patient positioned on a conductive fabric belt and connected to non-MRI compatible anesthetic monitoring equipment. Additional shielding to reduce EMI is provided by the aluminum tabletop, visible underneath the supporting cushions, and a semi-cylindrical aluminum arch that effectively encloses the body.

Based on a previously described system (O’Reilly et al. 2019; O’Reilly et al. 2021; de Vos et al. 2021; Obungoloch et al. 2023), the LF MRI is a Halbach array of permanent magnets with a total length of 50 cm, clear bore diameter of 31 cm, and main magnetic field strength (B_0_) of 0.05 T. An open bore design facilitates patient positioning prior to scanning, with a small door at the back of the bore that allows for placement of the required anesthetic equipment (Figure 1A, B). Imaging was conducted using a cylindrical spiral-solenoid transmit/receive head coil with a diameter of 23  cm.

The LF MRI was situated in a room, unshielded from radiofrequency (RF), located in a hallway between the HF MRI and anesthetic induction area. To minimize electromagnetic interference (EMI), shielding was enabled by an aluminum tabletop and a semi-cylindrical flexible arch, effectively enclosing the body and reducing EMI, as demonstrated in previous studies (Figure 1C) (Webb and O’Reilly 2023).

In accordance with the approved ethical work protocol, the allotted additional anesthesia time to perform the LF MRI scans was 45 min, and gadoterate meglumine could not be re-administered. If patients were anesthetically stable, they were immediately transferred from the HF MRI to the LF MRI and positioned in sternal recumbency in a foam immobilizer trough. As the patient’s body can act as an EMI antenna, a conductive fabric belt (Multiwave Inc., Geneva, Switzerland) was positioned between the patient and the trough and aligned along the bore (Lena et al. 2025). This belt was connected to electrocardiogram (ECG) electrodes attached to the metacarpal/metatarsal pads of three limbs and subsequently grounded to the magnet’s ground plane. Once grounded, patients were temporarily disconnected from the anesthetic rebreathing system to position the head within the coil, and the rebreathing tube was threaded through the back of the magnet bore. A small towel was placed underneath the patient’s head as needed to improve vertical alignment within the coil. Once reconnected, patients were positioned within the magnet bore with central alignment of the neurocranium.

Since the magnetic field of the LF MRI is temperature dependent due to the use of NdFeB permanent magnets, prior to image acquisition, the resonance frequency (*f*_*0*_) was determined, followed by impedance matching of the coil using a network analyzer (Copper Mountain Planar TR 1300/1 network analyzer, Indianapolis, IN, USA). After tuning the coil with each patient, a noise power spectrum (NPS) was generated to evaluate for excess radiofrequency receiver noise. If the fitted noise peak was above a threshold close to Johnson noise, various methods were implemented to reduce EMI, such as repositioning the dog, improving surface area contact between the dog and conductive belt, or attaching additional ECG electrodes. Auto-shimming was performed next, followed by calibration of the power required for 90° and 180° RF pulses. A one-dimensional profile was acquired along each orthogonal projection to ensure the patient’s neurocranium was centrally positioned in the bore and to provide approximate head dimensions to guide the selection of an appropriate field of view (FOV). If patient re-positioning was necessary, all steps from NPS evaluation onwards were repeated.

Once pre-imaging preparations were complete, transverse 3D T1w and T2w TSE sequences were acquired. For ease of reference, these sequences are similarly referred to as T1w and T2w from this point forward. Due to the exploratory nature of the study, the LF MRI protocol evolved over time based on preliminary image assessment at the time of imaging. Therefore, not all patients received identical sequences due to real-time adaptive modification of acquisition parameters as necessary to improve image quality while staying within the limitations of the ethical work protocol.

### 2.4. Image analysis

The MRI studies from all patients were processed to produce images in anonymized DICOM format with available sequences from each patient classified by system and protocol into four groups: (1) LF T1w, (2) LF T2w, (3) HF T1w, and (4) HF T2w. Within each group and per scoring session, the sequences were assigned a randomized numerical identifier (https://www.random.org/lists/). All sequences were retrospectively and independently scored on two separate occasions, 3–6 weeks apart, by two observers: E.B. (second-year ECVDI resident) and S.M. (board-certified radiologist of the American College of Veterinary Radiology). Observers were blinded to the patient’s signalment and reported clinical signs. The order of evaluation per scoring session was as follows: LF T1w, LF T2w, HF T1w, and HF T2w. The LF studies were assessed first to avoid any bias related to the identification of abnormalities in the HF studies. For the LF studies, a maximum of 7 patients per day was established to avoid decision fatigue. As both observers were experienced with studies acquired from the HF system, the number of patients evaluated per session was up to the observer's discretion. All images were evaluated in DICOM format on a radiological workstation with medical-grade monitors using dedicated software (RadiAnt DICOM Viewer, Medixant, Poznań, Poland; Horos, Nimble Co LLC, Purview, Annapolis, MD, USA).

A scoring protocol for the current study was developed by preliminary evaluation of one of the acquired LF T1w sequences and based on the observers’ experience with the previous pilot cadaver study. This amended protocol was broadly categorized into three domains: (1) scan quality, (2) anatomic feature identification, and (3) pathology.

#### 2.4.1. Scan quality

Each sequence was first assessed to determine whether the signal intensity of the cerebrospinal fluid (CSF), relative to cerebral parenchyma, was appropriate for the sequence: hypointense for T1w and hyperintense for T2w sequences. Sequences that produced inappropriate CSF signal intensity were excluded from the remainder of the study, along with the corresponding paired data on the other system.

Additionally, a structured semi-quantitative image quality scoring system was designed in order to develop a more objective means of assessing image quality for clinical usability. A total of six features were assessed using a three-point ordinal scale: noise, patient position, other artifacts (excluding noise, e.g. fold-over), regional anatomy distinction, grey/white matter (GWM) distinction, and CSF-parenchyma distinction. Noise was specifically evaluated separately from other artifacts as the 0.05 T system was located in an unshielded environment. Grading of noise, patient position, and artifacts was defined as 1 = marked limitations for clinical use; 2 = partial limitations for clinical use; 3 = no limitations for clinical use. Regional anatomy distinction was dependent on the delineation of the forebrain, midbrain, and hindbrain and was defined as 1 = one region well-defined and clearly delineated; 2 = two regions well-defined and clearly delineated; 3 = all regions clearly delineated. GWM distinction was assessed throughout the forebrain and defined as 1 = no visible differentiation; 2 = partially visible or ill-defined; 3 = well-defined and distinct. CSF-parenchyma distinction was evaluated at the dorsal horn of the lateral ventricles and defined as 1 = no distinction; 2 = both dorsal horns ill-defined, poorly delineated, or only one dorsal horn well-defined; 3 = both dorsal horns well-defined and clearly delineated.

#### 2.4.2. Anatomic features

The following 15 anatomic features were scored: lateral ventricles (LV), third ventricle (TV), mesencephalic aqueduct (MA), fourth ventricle (FV), optic nerve in canal or canal itself (CN2), falx cerebri (‘Falx’), internal capsule and corona radiata (WM), corpus callosum (CC), pituitary gland (PG), caudate nuclei (‘Caud’), thalamus (‘Thal’), midbrain, pons and medulla oblongata (‘CdBrainstem’), cerebellar lingula or nodulus (‘LingNod’), and cerebellar vermis (‘Vermis’). For the purpose of absolute visual grading characteristic (VGC) analysis, each feature was scored on a three-point ordinal rating scale, briefly defined as follows: 1 = not visible, 2 = partially visible, poorly delineated, and 3 = visible and well-defined. The individual rating definitions for each anatomic feature are available in the Supplementary Materials. Identification of features was aided by various anatomical and MRI references (Wisner and Zwingenberger 2015; Mai 2018; Hermanson and de Lahunta 2019; de Lahunta et al. 2021; Jacqmot et al. 2022).

#### 2.4.3. Pathology

The presence of pathology was determined via a binary visibility score as 1 = present or suspected and 2 = absent or not seen. If pathology was present, a brief description was made documenting the suspected lesion. For the purposes of this study, abnormalities of the ventricular system that are typically considered clinically incidental, such as breed-related ventriculomegaly, were scored as ‘1’ (Mai 2018).

### 2.5. Statistical analysis

Statistical analyses were performed using commercially available software (Microsoft Excel Version 2502, Microsoft; RStudio Version 2023.03.0 + 386, Posit). If applicable, a *p*-value < 0.05 was considered significant for relevant analyses. Descriptive statistics were used for patients’ sex, age, and weight, presented as median and interquartile range (IQR; 25th–75th percentile).

Inter- and intra-observer reliability of all scored features was assessed per system-sequence group with a Gwet’s agreement coefficient (κ_AC_) with linear weighting, which provides more stable reliability in the presence of high agreement or skewed distributions (Gwet 2008). Results are reported with percentage agreement (P_a_), 95% confidence interval (CI), and probabilistic benchmark as suggested by Landis and Koch (1977): < 0.00, poor; 0.00–0.20, slight; 0.21–0.40, fair; 0.41–0.60, moderate; 0.61–0.80, substantial; 0.81–1.00, almost perfect agreement. When a single scoring category was assigned to all observations, a Gwet’s AC kappa was not calculated.

Per sequence type, cross-system evaluation of the interobserver agreement results was used to determine which features were included for further analysis. This was done via a 3-tiered approach: Tier 1 = automatic inclusion, κ_AC_ > 0.6 and/or 100% agreement with a single scoring category for both systems; Tier 2 = conditional inclusion, κ_AC_ = 0.4–0.6 for at least one system; and Tier 3 = automatic exclusion, κ_AC_ < 0.4 for either system or calculated CI includes negative values. To include a Tier 2 feature, at least one of the following conditions had to be met: (A) the upper CI limit of the lower κ_AC_ value was >0.60; (B) the κ_AC_ value across both systems was 0.4–0.6.

The included features were weighed equally and summed to generate composite scores for scan quality and anatomy, presented as median (IQR; 25th–75th percentile). The relationship between composite scan quality and anatomy was assessed via Spearman’s rank correlation coefficient (*ρ*) with 95% CI and the following interpretation for correlational strength: <0.3, poor; 0.3–0.59, fair; 0.6–0.79, moderate; >0.8, very strong (Chan 2003).

Lastly, exploratory absolute VGC analysis comparing the LF and HF systems was performed, per sequence type, for the composite anatomy scores and each included feature. This is a non-parametric, rank-invariant method for assessing image quality based on the identification of clinically relevant anatomical structures. Similar to receiver operating characteristic (ROC) analysis, a VGC curve is generated based on the rating distributions of a test condition, the LF system, along the y-axis, and a reference condition, the HF system, along the x-axis. Calculation of the area under the curve (AUC_VGC_) gives an indication of the LF system’s performance relative to the HF. If the AUC_VGC_ was not statistically different from 0.5, this indicated comparable performance between systems (Båth and Månsson 2007; Båth 2010; Ludewig et al. 2010; Tesselaar et al. 2016; Precht et al. 2019). Due to the exploratory nature of the study, analysis was initially done across all included patient scans and subsequently done on ‘diagnostic quality’ scans only. A ‘diagnostic quality’ scan was defined by whether the median composite scan quality score across all observations for a patient was equal to or greater than the overall median composite scan quality score of the LF system. Analysis was performed using the VGCAnalyzer software (version 1.0, release 3) with a trapezoid VGC curve and random-reader model (Båth and Hansson 2016; Hansson et al. 2016; 2021). The results of the absolute VGC analysis were corroborated with per-feature assessment of system concordance, similarly using Gwet’s agreement coefficient.

## 3. Results

### 3.1. Patient demographics

A total of 23 client-owned dogs were prospectively enrolled, with exclusion of 1/23 (4%) due to failure to show for their scheduled appointment. In 1/23 (4%), troubleshooting of excessive noise led to lost scanning time and subsequent absence of comparative LF MRI sequences. Ultimately, 21 patients were included, consisting of 11 males (52.4%), with 4/11 intact (36.4%) and 7/11 (63.6%) castrated, and 10 females (47.6%), with 3/10 (30%) intact and 7/10 (70%) spayed. At the time of imaging, the median age was 7.4 years (IQR, 3.2–8.8 years) and the median bodyweight was 8.0 kg (IQR, 2.8–14.0 kg). Included cases represented 13 different breeds with 20/21 (95.2%) purebred and 1/21 (4.8%) mixed breed. Included breeds were French Bulldog (*n* = 4), Pomeranian (*n* = 4), Chihuahua (*n* = 2), Shih Tzu (*n* = 2), and one each of the following: American Cocker Spaniel, Bohemian Shepherd, English Cocker Spaniel, Jack Russel Terrier, Keeshond mix, Miniature Schnauzer, Pug, Shetland Sheepdog, and Toy Poodle.

The ASA status of the included patients was considered ASA-1 in 1/21 (4.8%), ASA-2 in 13/21 (61.9%), and ASA-3 in 7/21 (33.3%). Broadly classified, the clinical indication for imaging included abnormal behavior and/or neurological clinical signs (18/21; 85.7%), recheck after radiation therapy for a known intra-axial mass (2/21; 9.5%), or suspected pituitary-dependent hyperadrenocorticism (1/21; 4.8%). In decreasing order of frequency, reported clinical signs included abnormal behavior such as increased aggression (*n* = 10), circling (*n* = 6), cervical pain (*n* = 5), generalized seizures (*n* = 4), phantom scratching (*n* = 3), ataxia (*n* = 3), fly-biting (*n* = 2), pathologic nystagmus (*n* = 2), intermittent episodes of opisthotonus (*n* = 1), paresis (*n* = 1), head tilt (*n* = 1), and anisocoria (*n* = 1). The final imaging diagnosis concluded in the clinical radiological report is available in the Supplementary Materials.

### 3.2. Sequence parameters

For both systems, the sequence acquisition parameters are listed in Table 1, with acquisition information per patient available in the Supplementary Materials. Due to the variability in patients’ head shape and size, selection of an optimal FOV in the LF system required a trial-and-error approach to ensure complete coverage of the neurocranium while maintaining reasonable scan acquisition times. To help minimize acquisition times, fold-over artifacts were accepted as long as they fell outside the neurocranium. The number of averages in the LF MRI was adjusted based on the remaining allotted anesthesia time to acquire the sequences within the selected FOV.

**Table 1.** Acquisition parameters from both systems are presented as mode (mean), when applicable, except for in-plane field of view (FOV), presented as mode (range), and acquisition time, presented as mean (range). Acquisition time was dependent on the selected in-plane field of view (FOV) dimensions. Individual acquisition parameters are available in the Supplementary Materials.

	1.5 T		0.05 T	
	T1w (*n* = 21)	T2w (*n* = 17)	T1w (*n* = 21)	T2w (*n* = 17)
TR (ms)	694 (577)	6014 (5279)	400 (405)	2500 (2118)
TE_eff_ (ms)	14	100	17 (16)	128 (118)
ETL	1	16	6 (7)	15 (14)
In-plane FOV (mm)	99 x 99 (99–194)	100 x 100 (100–195)	120 x 120 (80–160)	120 x 120 (100–150)
Slice thickness (mm)	3	3	3	3
NSA	2	4	2	2
Acquisition time (min)	04:55 (03:00–07:07)	05:01 (02:26–08:59)	03:34 (02:02–05:06)	07:56 (05:04–11:41)

ETL = echo train length, NSA = number of averages, TEeff = effective echo time, TR = repetition time.

With the HF MRI, the type of coil used was dependent on patient size, with a dedicated 20-channel head and neck coil used in 8/21 (38.1%) and a dedicated 8-channel small extremity coil used in 13/21 (61.9%). A T1w and T2w sequence was acquired for 21/21 patients (100%).

With the LF MRI, the previously described coil was used with all patients (21/21, 100%). A T1w sequence was acquired in 21/21 patients (100%), while only 17/21 (81%) also had a T2w sequence. In the remaining 4/21 (19%) patients, a T2w sequence was not acquired due to difficulties with RF coil tuning (requiring the dog to be partially taken out of the coil), inadequate noise reduction (requiring repositioning of the conductive cloth or ECG pads), or both, any of which could result in lost scanning time.

#### 3.3. Scan quality

#### 3.3.1. Sequence CSF weighting

The LF T2w sequences of 5/17 patients (29.4%) had hypointense CSF signal intensity relative to the neuroparenchyma. The paired T2w sequences of these patients were excluded from further analysis. Based on the date of acquisition, these were the first five patients with an LF acquired T2w sequence with the following acquisition parameters, as mode (range): TR 1000  ms (1000–2000 ms), TE_eff_ 70  ms (70–84 ms), ETL 10 (10–14). To improve T2 weighting in subsequent patients, these parameters were increased to TR 2500  ms, TE_eff_ 128  ms (128–150 ms), and ETL 15.

The CSF signal intensity was as expected for all LF T1w, HF T1w, and HF T2w sequences (100%).

#### 3.3.2. T1w sequences

For T1w sequences, all scan quality features (6/6) were included with 100% interobserver agreement in the HF system for ‘noise’ and ‘distinction CSF-parenchyma’, and the remaining features had ‘moderate’ to ‘almost perfect’ interobserver reliability (κ: 0.43–0.98) across both systems. Intraobserver evaluation showed 100% agreement for both observers for ‘patient position’ and ‘distinction CSF-parenchyma’ in the HF system and for one observer (S.M.) for ‘artifacts’ in the HF system. Remaining features had ‘moderate’ to ‘almost perfect’ intraobserver reliability across both systems (κ_EB_: 0.54–0.93; κ_SM_: 0.42–0.95). In general, inter- and intra-observer reliability was lower in the LF system compared to the HF system (Supplementary Table 4).

**Figure 2.**
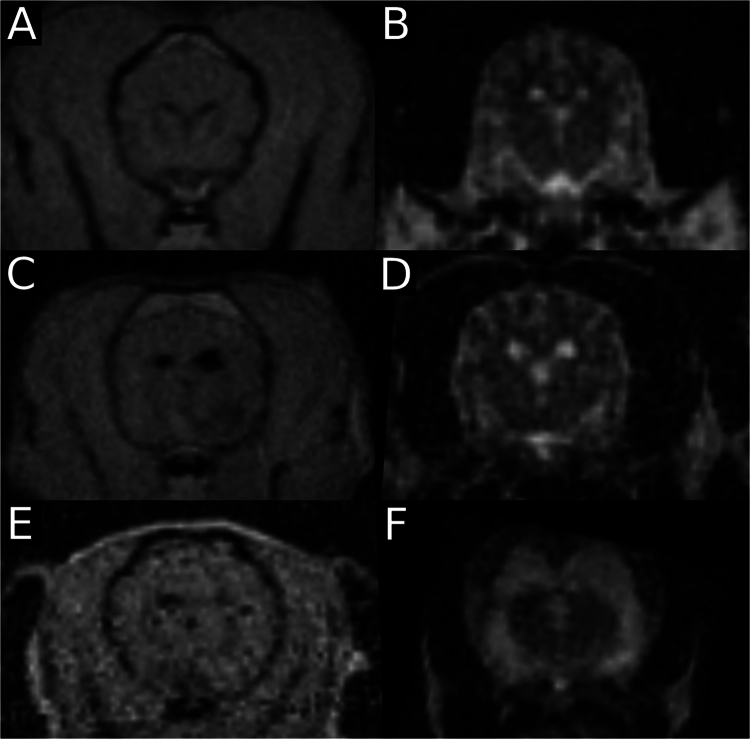
Examples of varying LF composite scan quality scores for transverse (A, C, E) T1w and (B, D, F) T2w sequences with scans (A–D) considered ‘diagnostic’ for their respective sequence with composite scores of 17, 18, 14, and 15, respectively. Scans (E and F) were considered ‘non-diagnostic’ with composite scores of 10 and 12.5, respectively. Dorsal is to the top and right to left of the images.

With the included features, the composite scan quality score could range from 6 to 18. The median score was 14 (IQR, 12–15) for the LF system and 17 (IQR, 16–17) for the HF system. A patient’s LF T1w sequence was considered ‘diagnostic quality’ if the median composite scan quality score across all observations was ≥14. Based on this cut-off, at least half of the patients (12/21) were considered to have ‘diagnostic quality’ scans. Figure 2 shows examples of varying LF scan qualities in both sequences.

#### 3.3.3. T2w sequences

For T2w sequences, all scan quality features (6/6) were included with 100% interobserver agreement in the HF system for ‘noise’, ‘patient position’, ‘artifacts’, ‘distinction regional anatomy’, and ‘distinction CSF-parenchyma’, and the remaining features had ‘moderate’ to ‘almost perfect’ interobserver reliability (κ: 0.41–0.91) across both systems. Intraobserver evaluation showed 100% agreement for both observers for ‘noise’, ‘patient position’, ‘artifacts’, ‘distinction regional anatomy’, and ‘distinction CSF-parenchyma’ in the HF system. One observer (S.M.) also had 100% agreement for ‘distinction grey-white matter’ in the HF system and ‘distinction CSF-parenchyma’ in the LF system. Remaining features had ‘moderate’ to ‘almost perfect’ reliability across both systems (κ_EB_: 0.53–1.00; κ_SM_: 0.48–1.00). In general, inter- and intra-observer reliability was lower in the LF system compared to the HF system (Supplementary Table 5).

With the included features, the composite scan quality score could range from 6 to 18. The median score was 15 (IQR, 14–16) for the LF system and 18 (IQR, 18–18) for the HF system. A patient’s LF T2w sequence was considered ‘diagnostic quality’ if the median composite scan quality score across all observations was ≥15. Based on this cut-off, at least half of the patients (7/12) were considered to have ‘diagnostic quality’ scans.

### 3.4. Anatomic feature identification

#### 3.4.1. T1w sequences

**Figure 3.**
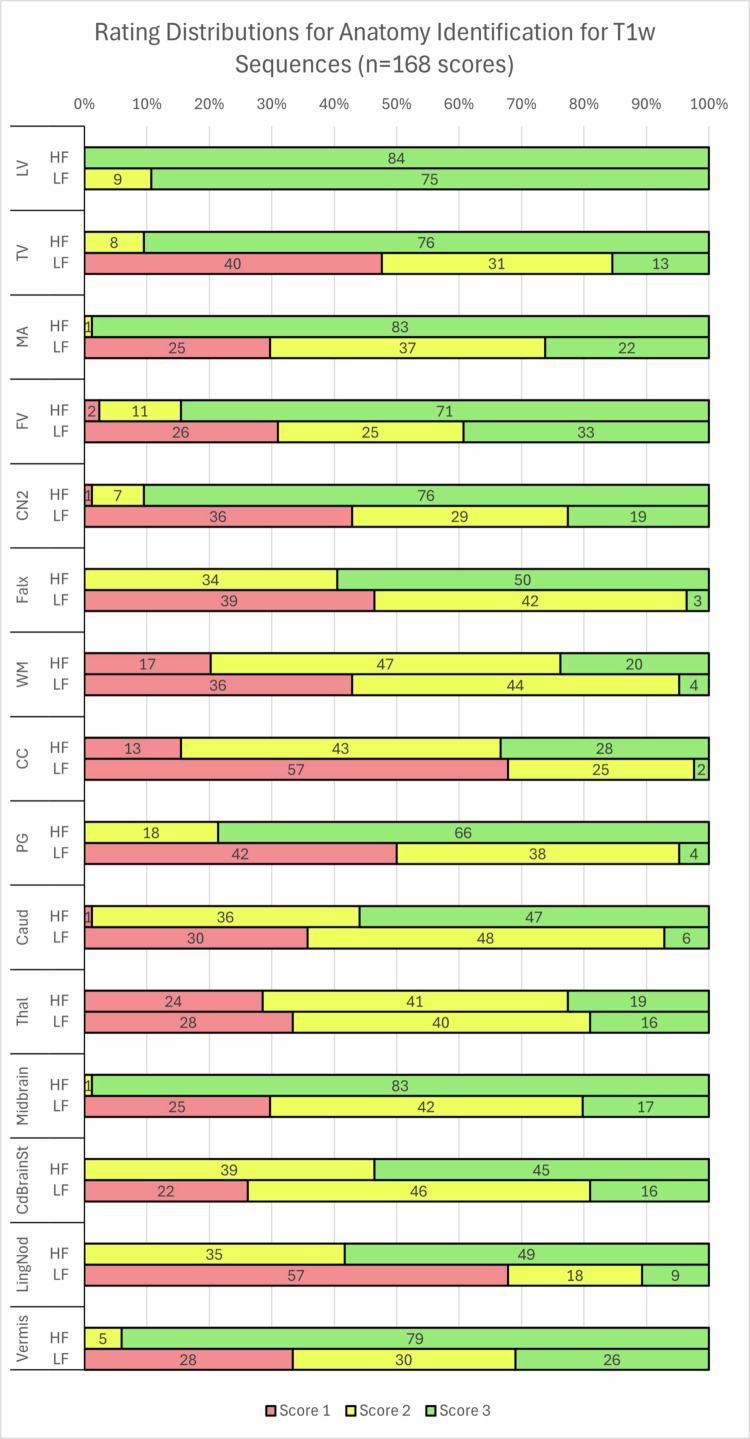
Combined stacked-clustered horizontal bar chart visualizing, per system, the rating distribution of 15 anatomic features in T1w sequences. LV = lateral ventricle, TV = third ventricle, MA = mesencephalic aqueduct, FV = fourth ventricle, CN2 = optic nerve in canal and/or optic canal, Falx = falx cerebri, WM = internal capsule and corona radiata, CC = corpus callosum, PG = pituitary gland, Caud = caudate nuclei, Thal = thalamus, CdBrainstem = medulla and pons, LingNod = lingula and/or nodulus, Vermis = cerebellar vermis.

Figure 3 shows the rating distribution for each anatomic feature in T1w sequences per system. Across both systems, the only feature assigned a score of 3 in more than half of the observations was the lateral ventricles, with 75/84 in the LF (89.3%) and 84/84 in the HF (100%). Features assigned a score ≥2 in more than half the observations with the LF system included the third ventricle (44/84, 52.4%), mesencephalic aqueduct (59/84, 70.2%), fourth ventricle (58/84, 69.0%), optic canal (48/84, 57.1%), falx cerebri (45/84, 53.6%), internal capsule/corona radiata (48/84, 57.1%), pituitary gland (42/84, 50%), thalamus (54/84, 64.3%), caudate nuclei (56/84, 66.7%), midbrain (59/84, 70.2%), caudal brainstem (59/84, 70.2%), and cerebellar vermis (56/84, 66.7%).

Based on the interobserver agreement, 4/15 features were excluded for further analysis (‘fourth ventricle’, ‘falx cerebri’, ‘caudal brainstem’, ‘lingula/nodulus’). Of the included 11/15 features, the majority had at least ‘substantial’ (*κ* > 0.6) intraobserver agreement across both systems for observers E.B. (LF 7/11, HF 11/11) and S.M. (LF 8/11, HF 9/11). In general, inter- and intra-observer reliability was lower in the LF system compared to the HF system (Supplementary Table 4).

With the included features, the composite anatomy score could range from 11 to 33. The median score was 20 (IQR, 18–23) for the LF system and 29 (IQR, 28–31) for the HF system. A ‘moderate’ positive correlation was seen between the composite scan quality and anatomy scores for both systems (Spearman’s *ρ* 0.69–0.71, CI 0.57–0.81, *p-*value < 0.001).

Table 2 shows the results of absolute VGC analysis for the T1w sequences. Across all scans, the LF system had comparable performance to the HF system for identification of the lateral ventricles (AUC_VGC_ 0.446, *p*-value 0.103) and caudate nuclei (AUC_VGC_ 0.473, *p*-value 0.677). The system concordance of these features (Supplementary Table 6) was almost perfect for the lateral ventricles (*κ* 0.88, CI 0.798–0.964) and slight for the caudate nuclei (*κ* 0.10, CI -0.064–0.258). Additionally, the system concordance for the internal capsule/corona radiata was relatively higher (*κ* 0.29, CI 0.12–0.462) than that of the caudate nuclei, though the AUC_VGC_ was not suggestive of comparable performance of the LF system. This is an interesting finding, as the internal capsule/corona radiata can help delineate the region of the caudate nuclei.

**Table 2.** Absolute VGC analysis results for T1w sequences with AUC_VGC_, standard deviation (SD), CI (95%), and *p*-value reported.

Variable	All scans				‘Diagnostic’ scans			
	AUC_VGC_	SD	CI (95%)	*p*-value	AUC_VGC_	SD	CI (95%)	*p*-value
Composite Anatomy	0.025	0.019	(0.000, 0.074)	<0.001	0.047	0.035	(0.000, 0.132)	0.001
Individual Feature								
*Lateral ventricles*	0.446	0.032	(0.369, 0.500)	0.103*	0.447	0.040	(0.354, 0.500)	0.203*
*Third ventricle*	0.098	0.037	(0.031, 0.179)	<0.001	0.113	0.043	(0.037, 0.207)	<0.001
*Mesencephalic aqueduct*	0.136	0.046	(0.052, 0.232)	<0.001	0.191	0.069	(0.063, 0.333)	0.003
*Optic canal*	0.147	0.055	(0.048, 0.260)	<0.001	0.227	0.071	(0.094, 0.372)	0.005
*Internal capsule and corona radiata*	0.337	0.067	(0.205, 0.471)	0.019	0.459	0.087	(0.288, 0.630)	0.631*
*Corpus callosum*	0.187	0.077	(0.049, 0.354)	<0.001	0.212	0.097	(0.043, 0.417)	0.009
*Sella/Pituitary gland*	0.072	0.030	(0.022, 0.137)	<0.001	0.090	0.038	(0.026, 0.174)	0.001
*Caudate nuclei*	0.473	0.074	(0.330, 0.617)	0.677*	0.607	0.083	(0.424, 0.748)	0.161*
*Thalamus*	0.197	0.054	(0.092, 0.304)	<0.001	0.236	0.082	(0.098, 0.413)	0.006
*Midbrain*	0.106	0.044	(0.028, 0.196)	<0.001	0.178	0.064	(0.063, 0.313)	0.001
*Cerebellar vermis*	0.170	0.052	(0.071, 0.274)	<0.001	0.253	0.069	(0.118, 0.389)	0.004

^*^ Features where the AUC_VGC_ was not statistically different from 0.5, indicating equal performance between the systems.

**Figure 4.**
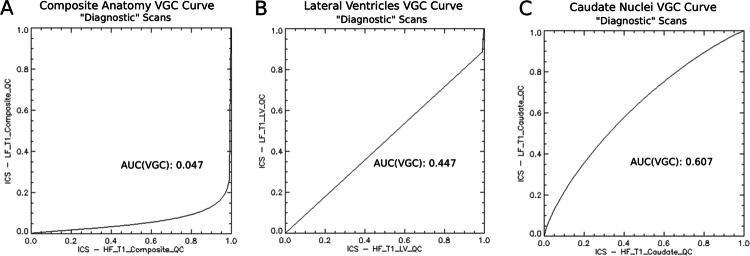
VGC curves for ‘diagnostic’ T1w scans for the (A) composite anatomy, (B) lateral ventricles, and (C) caudate nuclei.

With ‘diagnostic’ scans only, the AUC_VGC_ variably increases for the composite score and majority of features with the LF system showing comparable performance, relative to the HF, for the lateral ventricles (AUC_VGC_ 0.447, *p*-value 0.203), caudate nuclei (AUC_VGC_ 0.607, *p*-value 0.161), and internal capsule/corona radiata (AUC_VGC_ 0.459, *p*-value 0.631). Figure 4 shows the VGC curves from the ‘diagnostic’ quality scans for composite anatomy (4A), lateral ventricles (4B), and caudate nuclei (4C). For these features, the benchmark for system concordance improved only for the internal capsule/corona, going from fair to moderate, while the benchmark for the lateral ventricles and caudate nuclei remained the same. Additionally, the benchmark classification of system concordance improved from poor to fair for the optic canal, poor to slight for the mesencephalic aqueduct and midbrain, and slight to fair for the cerebellar vermis.

#### 3.4.2. T2w sequences

**Figure 5.**
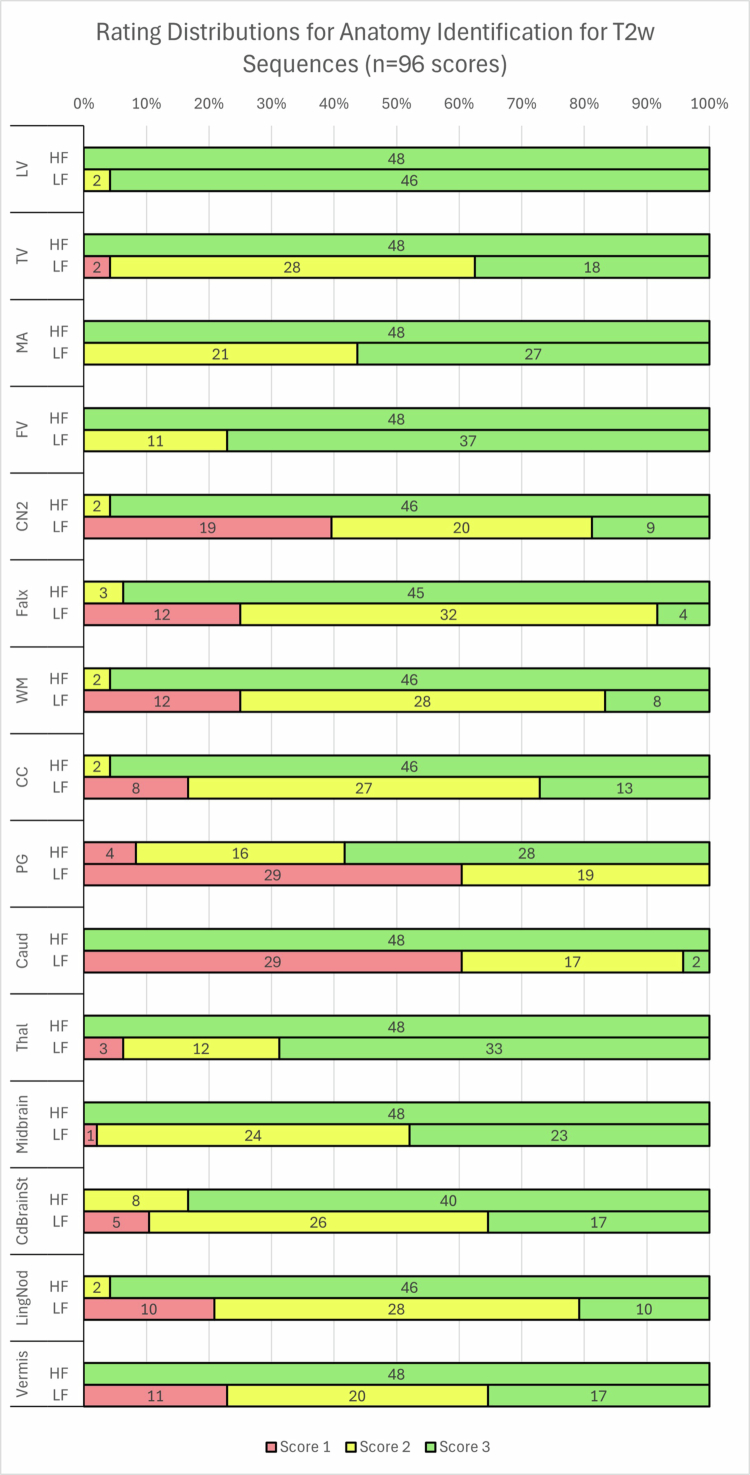
Combined stacked-clustered horizontal bar chart visualizing, per system, the rating distribution of 15 anatomic features in T2w sequences. LV = lateral ventricle, TV = third ventricle, MA = mesencephalic aqueduct, FV = fourth ventricle, CN2 = optic nerve in canal and/or optic canal, Falx = falx cerebri, WM = internal capsule and corona radiata, CC = corpus callosum, PG = pituitary gland, Caud = caudate nuclei, Thal = thalamus, CdBrainstem = medulla and pons, LingNod = lingula and/or nodulus, Vermis = cerebellar vermis.

Figure 5 shows the rating distribution for each anatomic feature in T2w sequences per system. Across both systems, the features assigned a score of 3 more than half of the observations were the lateral ventricles (LF 46/48 [95.8%]; HF 100%), mesencephalic aqueduct (LF 27/48 [56.3%]; HF 100%), fourth ventricle (LF 37/48 [77.1%]; HF 100%), and thalamus (LF 33/48 [68.8%]; HF 100%). Features assigned a score ≥ 2 in more than half of the observations with the LF system included the third ventricle (46/48, 95.8%), optic nerve/canal (29/48, 60.4%), falx cerebri (36/48, 75%), internal capsule/corona radiata (36/48, 75%), corpus callosum (40/48, 83.3%), caudate nuclei (45/48, 93.8%), midbrain (47/48, 97.9%), pons/medulla (43/48, 89.6%), lingula/nodulus (38/48, 79.2%), and cerebellar vermis (37/48, 77.1%).

Based on the interobserver agreement, 4/15 features were excluded for further analysis (‘third ventricle’, ‘corpus callosum’, ‘pituitary gland’, ‘vermis’). Of the included 11/15 features, the majority had at least ‘substantial’ intraobserver agreement (*κ* > 0.6) across both systems for observers E.B. (LF 6/11, HF 10/11) and S.M. (LF 9/11, HF 11/11). In general, inter- and intra-observer reliability was lower in the LF system compared to the HF system (Supplementary Table 5).

With the included features, the composite anatomy score could range from 11 to 33. The median score was 25 (IQR, 22–27) for the LF system and 33 (IQR, 33–33) for the HF system. For the LF system, a ‘moderate’ positive correlation was seen between the composite scan quality and anatomy scores with a Spearman’s *ρ* 0.74 (CI 0.58–0.86, *p-*value < 0.001). The HF system had a ‘fair’, positive correlation with a Spearman’s *ρ* 0.43 (CI 0.27–0.67, *p-*value 0.002), though this was likely lower due to the restricted range and low variability of the composite scan quality and anatomy scores in this group (Altman and Bland 1983; Schober et al. 2018).

Table 3 shows the results of absolute VGC analysis for the T2w sequences. Across all scans, the LF system had comparable performance to the HF system for identification of the lateral ventricles (AUC_VGC_ 0.479, *p*-value 0.310) with almost perfect agreement for system concordance (*κ* 0.96, CI 0.893–1.000; Supplementary Table 7). With ‘diagnostic’ scans only, the AUC_VGC_ variably increases for the composite score and majority of features with the LF system showing comparable performance, relative to the HF, for the lateral ventricles (AUC_VGC_ 0.464, *p*-value 0.303), mesencephalic aqueduct (AUC_VGC_ 0.325, *p*-value 0.175), fourth ventricle (AUC_VGC_ 0.378, *p*-value 0.121), thalamus (AUC_VGC_ 0.375, *p*-value 0.121), and caudal brainstem (AUC_VGC_ 0.381, *p*-value 0.224). For these features, the benchmark for system concordance improved for the mesencephalic aqueduct and caudal brainstem, both going from fair to moderate, while it remained the same for the lateral ventricles, fourth ventricle, and thalamus. Additionally, the benchmark improved from fair to moderate for the midbrain and poor to slight for the internal capsule/corona radiata.

**Table 3.** Absolute VGC analysis results for T2w sequences with AUC_VGC_, standard deviation (SD), CI (95%), and *p*-value reported.

Variable	All scans				‘Diagnostic’ scans			
	AUC_VGC_	SD	CI (95%)	*p*-value	AUC_VGC_	SD	CI (95%)	*p*-value
Composite Anatomy	0.004	0.006	(0.000, 0.021)	0.001	0.008	0.013	(0.000, 0.046)	0.006
Individual Feature								
*Lateral ventricles*	0.479	0.024	(0.417, 0.500)	0.310*	0.464	0.040	(0.357, 0.500)	0.303*
*Mesencephalic aqueduct*	0.282	0.072	(0.135, 0.417)	0.014	0.325	0.088	(0.143, 0.500)	0.175*
*Fourth ventricle*	0.384	0.054	(0.271, 0.479)	0.032	0.378	0.065	(0.250, 0.500)	0.121*
*Optic canal*	0.107	0.057	(0.017, 0.233)	<0.001	0.117	0.073	(0.000, 0.286)	0.010
*Falx cerebri*	0.068	0.038	(0.000, 0.153)	0.001	0.089	0.055	(0.000, 0.214)	0.006
*Internal capsule and corona radiata*	0.099	0.053	(0.010, 0.218)	<0.001	0.163	0.081	(0.036, 0.339)	0.014
*Caudate nuclei*	0.020	0.025	(0.000, 0.083)	0.001	0.035	0.043	(0.000, 0.143)	0.006
*Thalamus*	0.344	0.060	(0.208, 0.448)	0.019	0.375	0.067	(0.214, 0.482)	0.121*
*Midbrain*	0.240	0.069	(0.104, 0.375)	0.005	0.338	0.082	(0.161, 0.482)	0.062
*Caudal brainstem*	0.258	0.083	(0.102, 0.421)	0.014	0.381	0.098	(0.179, 0.571)	0.224^*^
*Lingula/Nodulus*	0.119	0.057	(0.021, 0.245)	<0.001	0.173	0.082	(0.036, 0.347)	0.010

^*^ Features where the AUC_VGC_ was not statistically different from 0.5, indicating equal performance between the systems.

#### 3.5. Pathology

For both sequences, cross-system evaluation of the interobserver agreement for the presence of pathology did not meet the inclusion criteria, and therefore, quantitative statistical analyses of pathological abnormalities were not performed (Supplementary Tables 4 and 5). While both sequences in the HF system had substantial agreement (κ: 0.67–0.7), agreement in the LF system was only fair for the T1w sequences (*κ* 0.33, CI 0.019–0.644) and slight for the T2w sequences (*κ* 0.08, CI −0.346–0.516).

**Figure 6.**
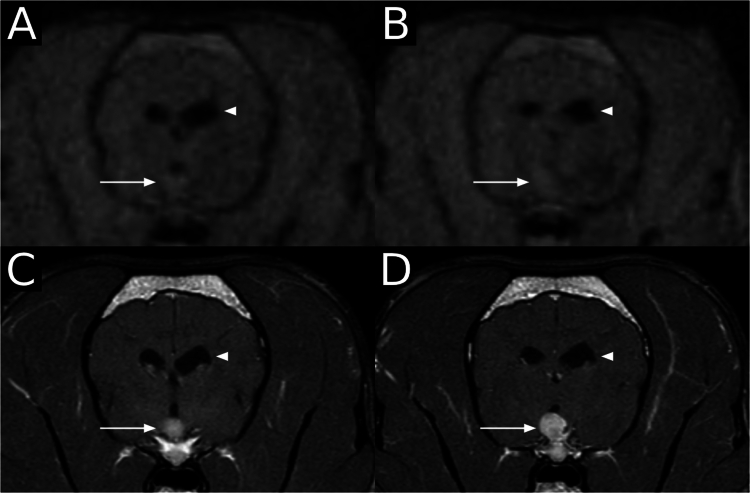
Transverse T1w images (3 mm slice thickness) at the level of the pituitary gland of patient #4 acquired with the (A, B) LF and (C, D) HF systems. Dorsal is to the top and right to the left of the images. In all images, a contrast-enhancing sellar mass with variably well-defined margins (arrow) and incidental breed-related ventricular asymmetry (arrowhead) are identified.

Assessment of the lesional descriptions showed consistent identification of ventricular abnormalities across all observations, including those considered clinically incidental, such as the asymmetric lateral ventricle size, in patients #3, 4, 8, 13, 14, 15, 17, 19, 20, 22. Additionally, a space-occupying lesion in the region of the pituitary gland was identified in all observations for patient #4 (Figure 6).

## 4. Discussion

In the current study, a previously developed LF canine cadaver brain scanning protocol was translated to *in vivo* application and evaluated in a diverse population of canine patients. The rating distributions of both LF sequences showed that 13/15 (86.7%) anatomic features were at least partially visible (score ≥2) in more than half of the observations, with relatively higher overall frequency in the T2w sequences. Notably, across all system-sequence combinations, the lateral ventricles were predominantly assigned a score of 3 (89.3%–100%) and, in the T2w sequences, this was also the case for the mesencephalic aqueduct (56.3%–100%), fourth ventricle (77.1%–100%), and thalamus (68.8%–100%). These findings were supported by absolute VGC analysis of the ‘diagnostic’ scans, which suggested that the LF system has comparable performance to the HF MRI for the identification of the lateral ventricles in both T1w and T2w sequences and, in T2w sequences, the mesencephalic aqueduct, fourth ventricle, and thalamus. Although lesion-based assessment of diagnostic performance was statistically limited in the current study, the consistent identification of ventricular abnormalities across all observations, in conjunction with the anatomical findings, is particularly relevant for the purposes of evaluating hydrocephalus.

The patients in the current study presented with clinical indications for a diagnostic MRI of the brain. Routine veterinary protocols for a diagnostic brain MRI include pre-contrast T1w and T2w sequences and a T1w sequence post-intravenous contrast administration (Wisner and Zwingenberger 2015; Mai 2018). As the HF MRI was performed first, the LF acquired sequences are post-contrast with an average time of 28 min (range 16–44 min) from contrast administration to the start of the LF protocol. The estimated mean half-life of gadoterate meglumine elimination, based on human pharmacokinetics, is 1.6 h; therefore, it was unlikely to have been fully eliminated by the time of LF image acquisition (Anon 2024). However, it is reported that the conspicuity of gadolinium-based contrast is reduced with decreasing field strengths and, in humans, it is recommended to double the standard dosage at low field strengths (<0.5 T) (Konar and Lang 2011; Hori et al. 2021; Chen et al. 2025). Unfortunately, the ethical work protocol did not allow for re-administration of contrast for the LF studies, and it was therefore expected that contrast enhancement would be less conspicuous. This was unlikely to impact identification, or lack thereof, for the majority of selected anatomic features. However, the pituitary gland is a structure that exhibits strong physiological enhancement, and a discrepancy in conspicuity of enhancement may have negatively affected its identification in the LF system (Mai 2018). Future LF studies, including post-contrast sequences, should consider an optimal dosage range for existing gadolinium-based contrast agents or explore other agents with stronger relaxivity effects, such as super-paramagnetic iron oxides (Liu et al. 2021; Chen et al. 2025).

**Figure 7.**
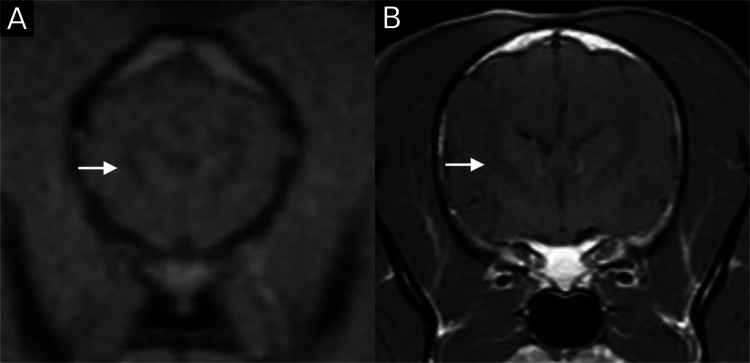
Example patients where the white matter tract, specifically the genu of the internal capsule (white arrow), was hypointense in both LF (A) and HF (B) acquired transverse T1w sequences. (A) Patient #7 had an unremarkable MRI of the brain. (B) Patient #9 had presumed bilateral otitis media and interna with focal meningitis adjacent to the right internal acoustic meatus. Dorsal is to the top and right to left of the images.

Interestingly, the VGC analysis results suggest that the LF T1w sequences had comparable performance to the HF system for identification of the caudate nuclei and major white matter tracts (internal capsule, corona radiata). These findings are suspected to be related to one another, with the internal capsule helping to delineate the region of the caudate nuclei rostrally. In fact, the number of observations with matched scores (e.g. 2 for both features) was 56/84 (66.7%) in the LF system and 38/84 (45.2%) for the HF system. Furthermore, the observers noted in multiple LF studies and a few HF studies that the white matter tracts, specifically the genu of the internal capsule, were hypointense relative to the gray matter in T1w (Figure 7). The T1 relaxation time of tissues is dependent on magnetic field strength, with marked shortening of T1 as field strength decreases, which subsequently reduces the difference between gray and white matter. In LF systems, this should theoretically diminish the ability to distinguish these features (Marques et al. 2019; Liu et al. 2021). For the LF system evaluated in the current study, O’Reilly and Webb (2022) have previously generated *in vivo* relaxation time maps that demonstrated that, compared to clinical high field strengths, the T1 values of gray and white matter were much shorter, while T2 values were similar. Additionally, the same study from O’Reilly and Webb (2022) showed that the T1/T2 values of CSF remained long, compatible with previous investigations demonstrating the independence of CSF T1/T2 values from magnetic field strength (Marques et al. 2019; Hori et al. 2021; Liu et al. 2021). Interestingly, multiple recent publications investigating the relationship between white matter microstructure and T1 relaxation suggest that the current understanding of T1 relaxation in complex physicochemical environments is incomplete. A variety of factors influencing the T1 relaxation of white matter have been identified, including water and iron content, compartmentation of water, degree of myelination, axon density and size, and fiber orientation relative to the main magnetic field (Stüber et al. 2014; van Gelderen et al. 2016; Schyboll et al. 2018; Manning et al. 2021; Kauppinen and Thotland, 2023). In the current study, the etiology of the hypointense internal capsule identified in the T1w sequences of some patients, namely in the LF system, is unknown and warrants further investigation in future studies.

Similar to the author’s previous cadaver study, the T2w sequences had improved contrast resolution compared to the T1w sequences, with a higher number of at least partially visible anatomic features. However, T2w sequences require longer acquisition times due to the necessity for longer TR times and to achieve an acceptable SNR, a relevant limiting clinical factor highlighted in the current *in vivo* study, with a mean acquisition duration of 07:56  min for T2w sequences compared to 03:34  min for T1w sequences. If all other parameters were similar, a general rule-of-thumb was that T2w sequences had double the acquisition time, for subjectively worse SNR, relative to the T1w sequence (Figure 4). Ideally, the NSA of T2w sequences could be increased to improve SNR, but this was often not feasible due to the limited anesthetic time. To improve the system for clinical veterinary application, optimization of the scanning protocol (e.g. more efficient FOV selection) could recuperate valuable scanning time while developments related to hardware or data processing (e.g. veterinary-designed RF coils, deep learning reconstruction algorithms to reduce noise) could improve SNR (Liu et al. 2021; Arnold et al. 2023; Webb and O’Reilly 2023). Further sequence development should prioritize achieving high SNR within reasonably short acquisition times.

The portability and low-cost features of the device are highlighted by the ability to rely on manual grounding and shielding to reduce EMI, as shown in previous studies (Lena et al. 2025). However, this can introduce significant variability, as demonstrated in the current study. Through trial and error, an optimal setup for consistently minimizing electromagnetic interference (EMI) was developed. Initial tests showed that certain monitoring devices, namely the monitoring ECG leads, significantly increased EMI levels despite appropriate electrical grounding of the system. After consultation with the supervising veterinary anesthesiologist, it was elected to remove the monitoring ECG leads during LF scanning, leading to noticeable improvements in EMI levels for cleaner, more reliable data collection. In cases where noise levels remained high despite these measures, additional troubleshooting steps were implemented, including patient repositioning within the bore and rechecking all grounding connections. Interestingly, in some patients, the pulse oximeter contributed significantly to noise levels, even if device positioning was similar to that of other patients where the noise levels were acceptable. A fully predictable pattern was not identified in the current study; therefore, if it was anesthetically feasible, the device was detached for the duration of scanning. The implementation of AI-based noise reduction software represents a viable route for mitigating this variability and should be considered for future *in vivo* studies (Liu et al. 2021).

To the authors’ knowledge, this is the first study to utilize the VGC Analyzer software (Båth and Hansson 2016; Hansson et al. 2016; 2021) in a veterinary setting, even though a short communication encouraging the use of VGC analysis to veterinary researchers was already published over a decade ago (Ludewig et al. 2010). The underlying assumption for the methodology of VGC analysis is that the ability to identify normal anatomy will be strongly correlated with the detection of pathological structures and, in human studies, it has been validated as an indirect means of assessing image quality (Båth and Månsson 2007; Båth 2010; Ludewig et al. 2010; Tesselaar et al. 2016; Båth and Hansson 2016; Hansson et al. 2016; Precht et al. 2019; 2021). A few key steps are necessary to perform VGC studies adequately, including defining the specific image criteria in an adequate scoring scale, the images themselves (e.g. number, quality), and the observers. Regarding these steps, limitations of the current study include the lack of defined image criteria for anatomical landmarks in veterinary radiology, an appropriate power calculation to determine the number of images and observers to include, and inconsistent LF scan quality (Ludewig et al. 2010; Precht et al. 2019). The limited number of images in the current study, particularly those of consistent ‘diagnostic’ quality, may have been partially compensated for by the fact that a large difference in image quality was expected between the systems, supported by the AUC_VGC_ of the composite anatomy score in both sequences. For future studies, it would be appropriate to have LF scans of consistent quality with better-defined criteria for anatomic landmarks, a larger patient sample size, and a minimum of 3 appropriately trained observers.

In conclusion, an LF MRI brain protocol, previously developed for canine cadavers, was successfully applied *in vivo* to a diverse population of canine patients. Evaluation of the LF acquired images, via comparison with paired HF studies, showed that a number of anatomic features can be at least partially identified. For the purpose of pediatric hydrocephalus neuroimaging, identification of different ventricular system compartments is the finding of greatest importance. Identification of the lateral ventricles in both sequences, and additional compartments in the T2w sequences, was supported by absolute VGC analysis using the VGC Analyzer software (Båth and Hansson 2016, Hansson et al. 2016; 2021). In order to assess the device’s true clinical applicability, future studies should prioritize generating consistent image quality in order to transition to lesion-based assessment of larger sample sizes with confirmed pathology.

## Supplementary Material

Supplementary Material Supplementary Material

SupplementaryTables_ElisabethBurgers.xlsx SupplementaryTables_ElisabethBurgers.xlsx
