# Supplementary material for: Canine brain imaging with a new low-field portable (0.05 T) MRI scanner: a pilot in vivo comparison to conventional 1.5 T
Source: Vet Q. 2026 Jul 14;46(1):2696026. doi: 10.1080/01652176.2026.2696026 (PMC13371494; doi:10.1080/01652176.2026.2696026)
Supplement: Supplementary Material [file TVEQ_A_2696026_SM9936.docx]

**Supplementary Materials**

**Anatomy Scoring Protocol**

| **Anatomic Feature** |
| --- |
| Lateral ventricles – when small, evaluate at dorsal horn   1. Not delineated at all 2. One well-defined or both ill-defined 3. Both well-defined and clearly delineated |
| 3^rd^ ventricle at the level of the interthalamic adhesion   1. Not visible 2. Partially visible, but not well-enough to measure the interthalamic adhesion height 3. Clearly delineates the interthalamic adhesion |
| Mesencephalic aqueduct at the midbrain   1. Not visible 2. Ill-defined / not well-defined delineated 3. Clearly visible and well-defined |
| 4^th^ ventricle   1. Not visible 2. Ill-defined, poorly delineated 3. Clearly visible and well-defined |
| Optic nerve in the canal from start of canal to optic chiasm   1. Not visible on either side 2. One side well-defined or both sides ill-defined 3. Both sides well-defined and clearly visible |
| Falx cerebri   1. Not visible at all 2. Ill-defined, poorly delineated when visible 3. Well-defined and clearly delineated across entire length |
| Major white matter tracts (internal capsule + corona radiata)   1. Not visible 2. Ill-defined over multiple slices or mixed visibility 3. Majority well-defined over multiple slices |
| Corpus callosum   1. Not visible 2. Ill-defined over multiple slices or mixed visibility 3. Majority well-defined over multiple slices |
| Pituitary gland   1. Not clearly delineated 2. Ill-defined PG margins, unable to measure for P:B ratio 3. Clearly delineated PG, could measure for P:B ratio |
| Caudate nuclei   1. Neither clearly delineated 2. One well-defined or both ill-defined 3. Both well-defined and clearly delineated |
| Thalamus at the teddy bear ears   1. Not well-defined or clearly delineated 2. One lobe well-defined or both lobes ill-defined, poorly delineated 3. Both lobes well-defined and clearly delineated |
| Midbrain   1. Not visible 2. Ill-defined, poorly delineated margins 3. Well-defined and clearly delineated |
| Caudal brainstem (pons/medulla)   1. Not clearly differentiated 2. Ill-defined, poorly differentiated margins 3. Well-defined with clearly delineated margins relative to cerebellum |
| Cerebellar lingula or nodulus   1. Not visible 2. Ill-defined, poorly delineated margins 3. Well-defined and clearly delineated |
| Cerebellar vermis   1. Not visible 2. Ill-defined, poorly delineated margins 3. Well-defined and clearly delineated |
